# Dishevelled stablisation at the cilium by RPGRIP1L is essential for planar cell polarity

**DOI:** 10.1186/2046-2530-1-S1-O21

**Published:** 2012-11-16

**Authors:** S Schneider-Maunoury, A Mahuzier, HM Gaudé, I Anselme, F Silbermann, M Leroux-Berger, M Montcouquiol, S Saunier, C Vesque

**Affiliations:** 1 CNRS UMR7622, Université Pierre et Marie Curie, France; 2 INSERM U983, Hôpital Necker-Enfants Malades, France; 3 INSERM U862, Université Bordeaux 2, France

## 

Cilia are involved in planar polarity in different systems but the mechanisms by which they influence the polarization process are unclear [1]. In order to clarify this issue, we investigated the function of the ciliary gene *Rpgrip1l* (*Ftm/NPHP8/MKS5*) in the mammalian cochlear sensory epithelium and in the zebrafish floor plate. We and others have previously shown that mutations in the human *RPGRIP1L* gene cause Meckel and Joubert type B syndromes [2]. The Rpgrip1l protein is localised at the ciliary transition zone and is required for transduction of the Hh/Gli pathway [3]. Our recent work has shown that Rpgrip1l patterns the telencephalon via the regulation of Gli3 proteolytic cleavage [4]. Here we show that in both the mammalian cochlear sensory epithelium and the zebrafish floor plate, Rpgrip1l is required for correct positioning of the basal body along the planar polarity axis. Our results strongly suggest that Rpgrip1l is essential for stabilizing the adaptor protein dishevelled at the basal body and/or cilium. Finally, we demonstrate that, in the zebrafish floor plate, the function of Rpgrip1l in basal body positioning is mediated by dishevelled. We propose that Rpgrip1l participates in a protein complex required for stabilizing dishevelled at the cilium, and that this stabilization is essential for asymmetric localization of the basal body along the planar polarity axis.
